# Supplementary material for: Patterns of Intron Gain and Loss in Fungi
Source: PLoS Biol. 2004 Nov 30;2(12):e422. doi: 10.1371/journal.pbio.0020422 (PMC532390; doi:10.1371/journal.pbio.0020422)
Supplement: Table S1 — Also available at http://genes.mit.edu/NielsenEtAl/. (4.3 MB ZIP). [file pbio.0020422.st001.zip › NielsenEtAl/html/689.html]

AN4209.1.NCU00025.1.MG06336.1.FG05384.1


```
 CLUSTAL W (1.82) Multiple Sequence Alignments - Introns Inserted


Sequence 1: MG06336.1	418 aa
Sequence 2: FG05384.1	414 aa
Sequence 3: NCU00025.1	418 aa
Sequence 4: AN4209.1	402 aa
Alignment Length: 440 aa
Number Identitical Residues: 183 aa
Alignment Score (without introns) 9449


MG06336.1 	----MASRAMVPFLVAMMLLTG0----------~DNQCVRNCE-DPDPKKRVHFEQPVIQ
NCU00025.1	MASIIPARAIIPFLVGMMLLTG~VCNTLLTKYQ0DNQCVRNCD-DPDPLKRKHFEQPVIQ
FG05384.1 	----MASRSLIPFLVAMMLLTG~VCNTLLTKYQ0DNQCVRNCDPDDNPRKRAFFNQPVLQ
AN4209.1  	----MASKAVIPFLVTMMLVTG~VCNTILNKYQ0DMQCVRNCD-SPDPKDHHLFEQPVIQ
          	    :.:::::**** ***:**  ..:  .. . * ******: . :* .:  *:***:*

MG06336.1 	T~AQMFVGEMGCWLVIGLMSLRERYMARK-QAAGYEAVRS----SDDVP-DADDASVHST
NCU00025.1	T~LQMFVGEMGCWLVVGIMSLWSRYVSKS---TTYERINNRAGEDEDTT-VPDDASIRSH
FG05384.1 	T~AQMFIGEMGCWLVVGLMALWRRFKGSAPEQRGYQAVNT----EEGAD-STAEPEANDR
AN4209.1  	T2IQMFIGEMGCWLVVLMSHLYKRHLAPRLFQDQSPLLAGGYRPVNVVDGDDEDQTIEGH
          	*  ***:********: :  *  *. .          :       : . .   :   .. 

MG06336.1 	TALNGTGAGKPVHAHDSMLAGIRVFLLAIPAICDICGTTLMNAGLLMVAAS~IYQMTRGA
NCU00025.1	TPLTDSTASSKYDG-PSILRGWRVVLLSLPAICDICGTTLMNAGLLLVAAS~IYQMTRGA
FG05384.1 	TKLLHTGP--------SVLRGYRVTLLALPAICDICGTTLMNAGLLMVAAS~IYQMTRGA
AN4209.1  	NDNIDPSKPLRLPDGRIPLRGLRTFLLAAPACCDIAGTTLMNVGLLFVAAT1LCVVVLG-
          	.    .            * * *. **: ** ***.******.***:***: :  :. * 

MG06336.1 	LVLFVGLFSVVFLRRRLHLFQWLSLVGVVTGVAIVGLAGAIYPDKAKPGSPHSEVVPTST
NCU00025.1	LVLFVGIFSVVFLRRKLYAFQWLSLVGVMVGVALVGLAGAIQPDQKHPSA----------
FG05384.1 	LVLFVGLFSVIFLKRHLHLFQWLSLVGVVLGVAVVGLAGAIWPDEKPEGS-------IGT
AN4209.1  	-VALVGLAGALFGDNQGHDITQETVAAAVRSATTEVGATAQTPE----------------
          	 * :**: ..:*  .: : :   ::...: ..:    * *  *:                

MG06336.1 	PFQAASTAEGGLDANALRAVVGVLLIAGAQIFTATQFVVEEWLLERSTIEPIRVVGWEGI
NCU00025.1	--TTTTTADVGAD--ALKVIIGVLMIAGAQIFTATQFVLEEYILERSTIEPIRVVGWEGL
FG05384.1 	P-EEDSLADGGLS-DATRAIVGVLLIAGAQIFTATQFVLEEWLLERSSIEPLRVVGWEGI
AN4209.1  	---------------AVRAVVGVLLIAAAQIFTASQFVLEEWILENYAMEPLQVVGWEGI
          	               * :.::***:**.******:***:**::**. ::**::******:

MG06336.1 	FGFSVTVLGMIVMHLAIGRTETGKYGYFDMVEGWRQMVENPRVLISSVLIMISIG2GFNF
NCU00025.1	FGFTVTLLGMVILHFAIGRTEAGRYGPFDMVEGWRQFWEYKPVFISSVLIMISIG2GFNF
FG05384.1 	FGFSFTLLGMLILHAAIGSTDAGRYGYFDIVEGWRQMTENKQVLFSSFLIMISIG2GFNF
AN4209.1  	FGFSVTVIGSIILYLGVGRTEAGRYGYFDAREGWHQVFTHKAVAISSVLIMISIG2GFNF
          	***:.*::* :::: .:* *::*:** **  ***:*.     * :**.******* ****

MG06336.1 	FGLSVTRTVSATSRSTIDTCRTLFIWIVSLGLGWETFKWLQVLGFGFLVYFTFLFNGIVQ
NCU00025.1	FGLSVTRSVSATSRSTIDTCRTLFIWMVSLGLGWETFKWLQVIGFGLLVYFTFLFNGIVE
FG05384.1 	FGLSVTRSVSATSRSTIDTCRTLFIWVVSLGLGWETFKWLQILGFALLVYFTFLFNGIVQ
AN4209.1  	FGLSVTRTVSATSRSTIDTCRTLFIWLVSLGLGWETFKWLQVAGFALLVYGTFLFNDIVR
          	*******:******************:**************: **.:*** *****.**.

MG06336.1 	PPFKFLQV-DEEIEELLPEEPVEHI
NCU00025.1	PPFEFLRV--SQVEELLPEEPIEHN
FG05384.1 	PPFEFLKVPDEEIEELLPEDPIEHR
AN4209.1  	PPLKACLPREGRGRVLLPEDPIEHN
          	**::     . . . ****:*:**
```
